# Mechanisms of leukocyte distribution during sepsis: an experimental study on the interdependence of cell activation, shear stress and endothelial injury

**DOI:** 10.1186/cc9322

**Published:** 2010-11-08

**Authors:** Annette Ploppa, Volker Schmidt, Andreas Hientz, Joerg Reutershan, Helene A Haeberle, Boris Nohé

**Affiliations:** 1 Department of Anesthesiology and Intensive Care Medicine, Tuebingen University Hospital, Eberhard-Karls University, Hoppe-Seyler-Str. 3, Tuebingen, 72076, Germany; 2 Department of Gastroenterology, Leonberg Hospital, Rutesheimer-Str. 50, Leonberg, 71229, Germany

## Abstract

**Introduction:**

This study was carried out to determine whether interactions of cell activation, shear stress and platelets at sites of endothelial injury explain the paradoxical maldistribution of activated leukocytes during sepsis away from local sites of infection towards disseminated leukocyte accumulation at remote sites.

**Methods:**

Human umbilical venous endothelial cells (HUVEC) and polymorphonuclear neutrophils (PMN) were activated with lipopolysaccharide at 100 and 10 ng/ml to achieve adhesion molecule patterns as have been reported from the hyper- and hypo-inflammatory stage of sepsis. To examine effects of leukocyte activation on leukocyte-endothelial interactions, activated HUVEC were perfused with activated and non-activated neutrophils in a parallel plate flow chamber. Adhesion molecule expression and function were assessed by flow cytometry and blocking antibodies. In a subset of experiments the sub-endothelial matrix was exposed and covered with platelets to account for the effects of endothelial injury. To investigate interactions of these effects with flow, all experiments were done at various shear stress levels (3 to 0.25 dyne/cm^2^). Leukocyte-endothelial interactions were analyzed by videomicroscopy and analysis of covariance.

**Results:**

Activation of neutrophils rendered adhesion increasingly dependent on shear stress reduction. At normal shear stress, shedding of L-selectin decreased adhesion by 56%. Increased rolling fractions of activated PMN at low shear stress revealed impaired integrin affinity despite numerical up-regulation of CD11b. On sub-maximally activated, intact HUVEC shear stress became the prevailing determinant of adhesion. Presence of a platelet-covered injury with high surface density of P-selectin was the strongest variable for adhesion. When compared to maximally activated HUVEC, platelets increased neutrophil adhesion by 2.7-fold. At sub-maximal activation a 10-fold increase was observed (*P *< 0.05 for all).

**Conclusions:**

L-selectin shedding and integrin dysfunction render leukocyte adhesion increasingly susceptible to shear stress and alternative adhesion receptors. In combination, these effects inhibit recruitment to normally perfused sites with intact endothelium and favor maldistribution towards sites with compromised perfusion or endothelial injury.

## Introduction

Directing leukocytes to local sites of infection is a crucial part of the innate immune response. While intravascular shear forces prevent relevant leukocyte adhesion in a healthy individual, increased concentrations of microbial toxins and pro-inflammatory mediators induce upregulation of endothelial adhesion molecules in inflamed tissue, resulting in a targeted accumulation of leukocytes at the site of infection [1]. Initially, selectin-dependent interactions overcome postcapillary shear stress, enabling capture and rolling of leukocytes on the activated endothelium. Selectin-interactions and local chemokines then activate leukocyte integrins such as lymphocyte function antigen-1 (LFA-1, CD11a/CD18) and macrophage antigen-1 (MAC-1, CD11b/CD18). Local activation of integrins favours interactions with endothelial counter-receptors, such as intercellular adhesion molecule-1 (ICAM-1), resulting in firm adhesion [1].

In contrast to local inflammation, systemic sepsis is characterized by profound leukocyte activation throughout the circulation [2,3]. Because organ damage is attenuated by inhibiting leukocyte-endothelial interactions, systemic leukocyte activation and disseminated leukocyte adhesion are regarded essential for septic organ dysfunction [4-7]. In the last few years this traditional assumption has been challenged by the finding of an impaired chemotaxis and decreased rather than increased leukocyte recruitment to local sites of infection in septic individuals despite persistent upregulation of leukocyte integrins [2,3,8-10]. Moreover, it has been recognized that systemic hyper-inflammation often turns into hypo-inflammation with immunosuppressive cytokine-profiles such as increased ratios of interleukin (IL)-10 and tumor necrosis factor (TNF)-α [11-13]. Similar to the phenomenon of endotoxin tolerance, endothelial sensitivity to microbial toxins becomes altered and endothelial cell adhesion molecule expression is impaired [14-17]. Paradoxically these changes do not seem to protect patients from the development of endothelial cell damage and leukocyte-related organ dysfunction since they are most pronounced in those with poor prognosis [12,13]. To provide more insight into the mechanisms that contribute to these apparently paradoxical findings, we investigated the following questions in a flow chamber model with lipopolysaccharide induced inflammation.

First, does systemic leukocyte activation increase or impair leukocyte recruitment to activated endothelium and what are the mechanisms during the different stages of inflammation? Second, if targeted leukocyte recruitment to locally activated endothelium is impaired, are there mechanisms that favour disseminated leukocyte accumulation at the same time? Third, given that later sepsis is characterized by immunosuppression, endothelial cell damage and organ dysfunction, are there mechanisms, independent of the physiological immune response, that gain a leading role for the distribution of leukocyte accumulation?

## Materials and methods

### Endothelial cell culture and leukocyte separation

In compliance with the Helsinki Declaration on experimental research on humans and after obtaining ethical committee approval (local ethics committee, University of Tuebingen, reference numbers 315/99 and 69/2003-A) and informed consent, human umbilical venous endothelial cells (HUVEC) and polymorphonuclear neutrophils (PMN) were derived from human umbilical veins and citrated blood samples from healthy volunteers as previously described [18]. HUVEC were harvested by collagenase treatment (collagenase A 0.1%, Boehringer, Mannheim, Germany) and cultured in Endothelial Cell Growth Medium (EGM™, PromoCell, Heidelberg, Germany) on collagen-coated rectangular coverslips (Falcon Biocoat™, Becton Dickinson Labware, Bedford, MA, USA). Confluent HUVEC of the first passage were used for the experiments.

PMN were isolated by density gradient centrifugation at 1,700 rpm on a discontinuous Percoll gradient with 63% and 72% Percoll in buffer (Percoll, 1.130 g/ml; Amersham Pharmacia Biotech, Uppsala, Sweden). The bottom layer was collected and contaminating erythrocytes were removed by hypotonic lysis in 10% NH_4_Cl on ice. After washing, the PMN pellet was resuspended in cold Medium 199 (Sigma, St. Louis, MO, USA) supplemented with 50% fetal calf serum (Gibco, Mannheim, Germany) at 5 × 10^7^/ml. To avoid assay related activation of PMN during rewarming, we reconstituted the PMN pellet to 10^6 ^PMN/ml just before the adhesion assay in normoxic, room temperature Medium 199 only. Final rewarming to 37°C was achieved in the heatable flow chamber.

### Adhesion assay

**Figure 1:**
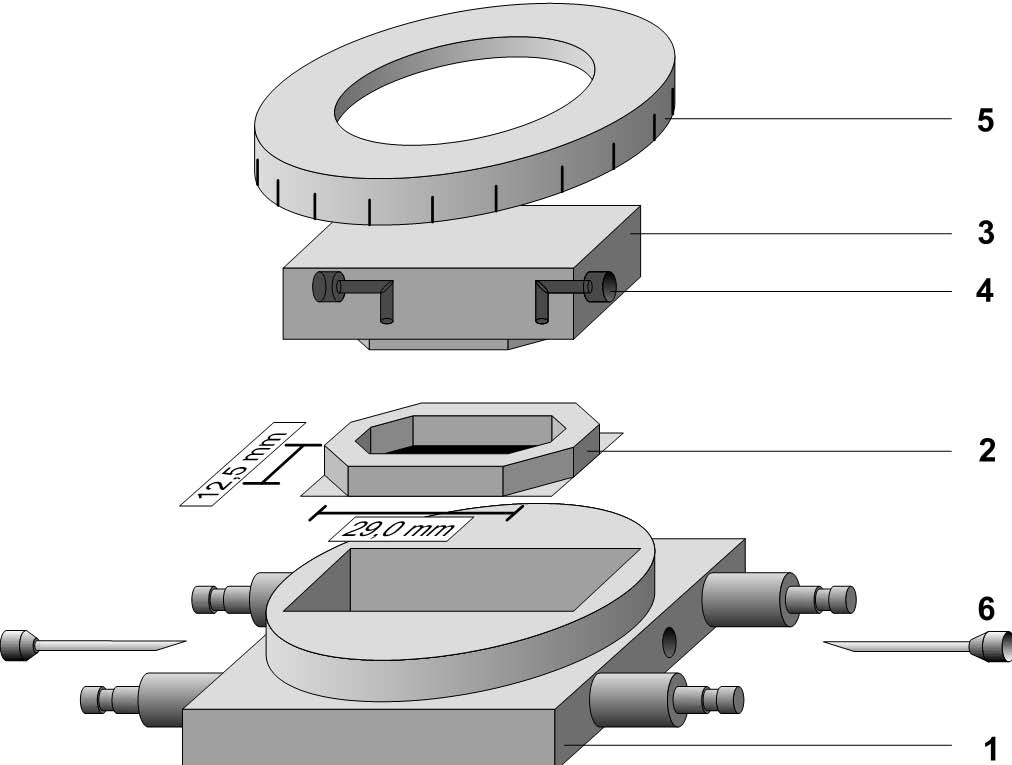
**Parallel plate flow chamber**. The flow chamber consisted of a heatable metal case **(1)**. The silicone-sealed coverslips **(2) **were placed in the middle. Using a transparent cover block **(3) **with a flow channel **(4) **and a scaled metal ring **(5)**, the chamber could be closed to a defined height leaving an inner chamber with a defined height of 0.2 mm. The tubing of the cell suspension was connected by a needle **(6) **to the inlet and outlet port of the transparent cover block (4). Temperature was controlled by temperature measurement within the metal case. Preliminary experiments showed that temperature of the metal block equaled with temperature of the perfusate within few seconds. For microscopy of the adhesion assay, the whole system was placed on an inverted phase-contrast microscope.

PMN adhesion to HUVEC was quantified in a parallel plate flow chamber with a laminar flow profile (Reynolds number <1, Figure 1) at 37°C as previously reported [18]. According to those shear forces that have been observed in postcapillary venules of normal and septic individuals we varied shear stress from 3 to 0.25 dyne/cm^2 ^[19-25].

PMN were perfused over HUVEC-containing coverslips for 10 minutes under different conditions of LPS-activation. Thereafter, PMN-adhesion was determined from 10 s video recordings of five different fields of view by phase contrast microscopy (20× objective; DMIRB, Leica, Bensheim, Germany). PMN were defined as rolling when traveling below 50% of the velocity of free flowing PMN in close proximity to the endothelium at the given shear stress [26]. A PMN, moving less than one cell diameter in 10 s was defined to be firmly adherent. To exclude sedimentation artefacts, we exposed the adherent PMN, stepwise, up to 32 dyne/cm^2 ^after the end of the adhesion experiment and measured cell detachment. Under this exposure >70% of the adherent PMN remained bound. As a measure for adhesion efficiency [27,28], the rolling fraction was calculated as: *(No. of rolling cells) × 100)/(No. of rolling cells + No. of firmly adherent cells). Mean rolling velocities were determined from more than 25 individual velocity profiles for each experimental condition as derived from customized software for image recognition (CellTracker, C. Zanke, University of Tuebingen, Germany).

Selectin function was determined at 2 dyne/cm^2 ^in presence of functional blocking monoclonal antibodies (mAb). PMN and HUVEC were incubated for 30 minutes prior to the adhesion assay with mAb against endothelial (E)-selectin (P2H3; Chemicon International, Temecula, CA, USA), leukocyte (L)-selectin (DREG-56; BD Biosciences Pharmingen, San Jose, CA, USA), platelet (P)-selectin (WASP12.2; Endogen, Woburn, MA, USA) or a nonspecific antibody (HP6069; BD Biosciences Pharmingen).

### Activation protocol modelling different stages of sepsis

By combining different conditions of neutrophil and endothelial activation, we intended to mimic patterns of adhesion molecule expression as they have been observed during local inflammation and different stages of sepsis-associated systemic hyper- or hypo-inflammation [1,2,8-10,29-31]. As detailed in Table 1, HUVEC were activated for four hours and PMN for 30 minutes with either 0 ng/ml, 10 ng/ml or 100 ng/ml LPS (026:B6 from *Escherichia coli*, Sigma), dissolved in Medium 199 supplemented with 20% fetal calf serum.

**Table 1:** Description of the different groups and their activation protocol

Group	Activation	Description
**HUVEC-/PMN-**	HUVEC 0 ng/ml LPS	Control (non-inflamed tissue)
	+ PMN 0 ng/ml LPS	
**HUVEC++/PMN-**	HUVEC 100 ng/ml LPS	Maximal local inflammation
	+ PMN 0 ng/ml LPS	
**HUVEC++/PMN ++**	HUVEC 100 ng/ml LPS	Maximal systemical inflammation in the hyper-inflammatory stage of sepsis
	+ PMN 100 ng/ml LPS	
**HUVEC+/PMN-**	HUVEC 10 ng/ml LPS	Submaximal local inflammation
	+ PMN 0 ng/ml LPS	
**HUVEC+/PMN +**	HUVEC 10 ng/ml LPS	Submaximal systemical inflammation in the hypo-inflammatory stage of sepsis
	+ PMN 10 ng/ml LPS	
**HUVEC++/PMN++/PLT**	HUVEC 100 ng/ml LPS	Maximal systemical inflammation and endothelial damage in the hyper-inflammatory stage of sepsis
	+ PMN 100 ng/ml LPS	
**HUVEC+/PMN+/PLT**	HUVEC 10 ng/ml LPS	Submaximal systemical inflammation and endothelial damage in the hypo-inflammatory stage of sepsis
	+ PMN 10 ng/ml LPS	

HUVEC, human umbilical venous endothelial cells; PMN, polymorphonuclear neutrophils; PLT, platelets; LPS, lipopolysaccharide.

The changes in adhesion molecule expression were determined by flow cytometry (FACSort™, Becton Dickinson, San Jose, CA, USA). Cells were gated using forward and side scatter properties and staining with saturating amounts of fluorochrome conjugated mAb against E-selectin, L-selectin (both from BD Biosciences), ICAM-1 (Immunotech, Marseille, France) and CD11b (Caltag, San Francisco, CA, USA). Matching isotype controls were used to define the setup of the instrument. Unintended PMN-activation during cell separation was ruled out by comparison of isolated PMN to leukocytes from whole blood.

### Activation protocol modelling endothelial injury

Distinct from true endothelial activation, severe sepsis leads to endothelial cell injury which is likely to persist even in the hypo-inflammatory stage [30,32] and results in platelet (PLT)-adhesion to the subendothelial matrix [33,34]. To account for PLT-PMN interactions under these conditions, we compared PMN-adhesion to activated HUVEC with PMN-adhesion to PLT-treated, injured HUVEC (Table 1) using a previously described model for endothelial injury [33]. By pipetting medium at high shear into the center of the coverslip an endothelial injury with exposure of the subendothelial matrix was created. To allow for platelet-matrix interactions, the coverslips were perfused with citrated whole blood at 20 dyne/cm^2 ^for five minutes prior to the PMN adhesion assay. Since platelet-matrix interactions are much more shear-resistant than leukocyte-endothelial interactions, this resulted in dense platelet accumulation at the site of injury without premature leukocyte adhesion. Before starting the PMN adhesion assay, the chamber was cleared from remaining blood by a thorough rinse with cell free medium. Then, the platelet-covered HUVEC were perfused with the PMN suspension at 2 to 0.25 dyne/cm^2^.

### Statistics

All experiments were carried out in quadruplicate. The medians of fluorescence intensity (MFI) were calculated from 5,000 single events by flow cytometry. An analysis of variance (ANOVA) was performed to determine whether adhesion molecule expression was influenced by LPS activation. Using an analysis of covariance (ANCOVA) and *post hoc *t-tests, we examined whether PMN activation (nominal effect), shear stress (continuous effect) or a combination thereof influenced PMN adhesion. Effects of platelets were analyzed accordingly (replacing PMN-activation by PLT-treatment). Effects of antibody blockade were examined by paired t-tests. Results of the adhesion assays are presented as means ± SEM. A *P*-value <0.05 after Bonferroni-Holm correction was considered significant. All analyses were performed using the statistical software JMP (SAS Institute Inc., Cary, NC, USA).

## Results

**Figure 2:**
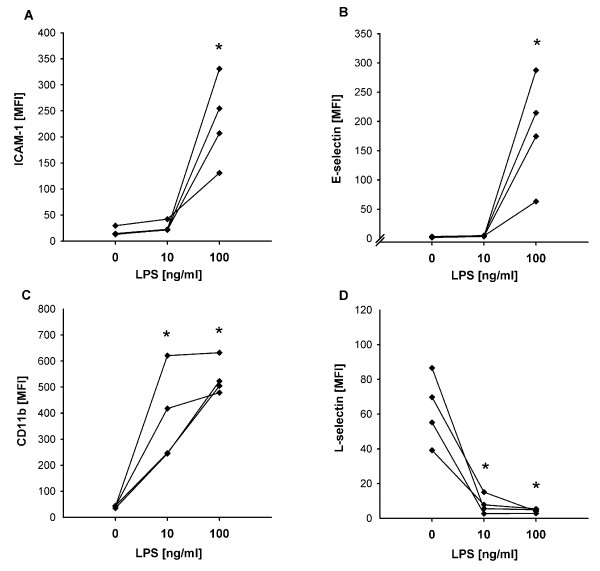
**Effects of different concentrations of LPS on the expression of adhesion molecules determined by flow cytometry**. **(a) **ICAM-1, **(b) **E-selectin, **(c) **CD11b, **(d) **L-selectin. Induction of E-selectin and ICAM-1 expression on HUVEC required maximal activation with LPS, whereas the sub-maximal activation induced a shedding of L-Selectin and increase of CD 11b-expression on PMN (* *P *< 0.01 vs. 0 ng/ml; ANOVA of logarithms).

When compared to non-activated controls (HUVEC-/PMN-), maximal LPS-activation with 100 ng/ml (HUVEC++/PMN++) resulted in maximal upregulation of E-selectin, ICAM-1, CD11b and complete shedding of L-selectin, comparable to systemic hyper-inflammation [10,29-31]. Similar to the hypo-inflammatory stage of sepsis [2,3,10-17], submaximal activation with 10 ng/ml still upregulated CD11b and downregulated L-selectin on PMN to the same degree as 100 ng/ml did, however, without having an effect on endothelial cell adhesion molecule expression (Figure 2).

### Effects of cell activation, shear stress and their interplay on PMN-HUVEC adhesion

**Figure 3:**
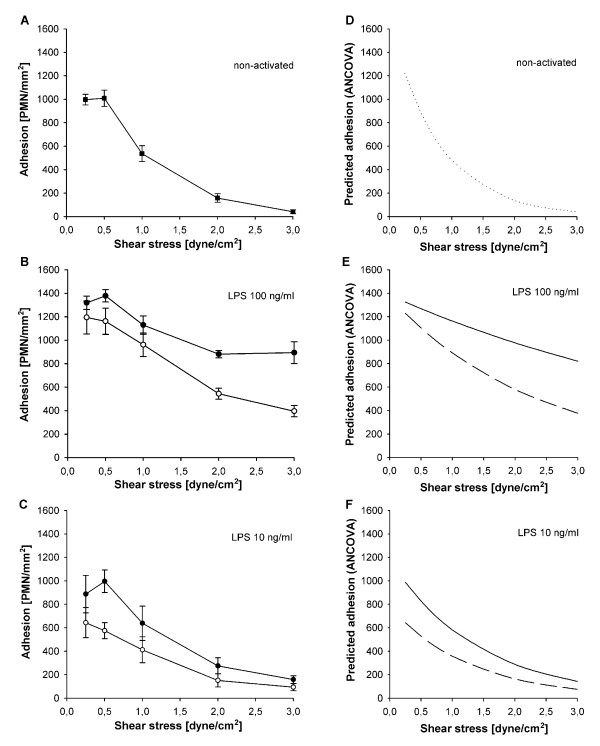
**Interdependent effects of shear stress and cell activation on PMN adhesion**. Adhesion of neutrophils under different activation protocols (mean ± SEM; *n *= 4), **(a) **non-activated controls, **(b) **activation with 100 ng/ml LPS and **(c) **activation with 10 ng/ml LPS. Blank symbols indicate activated PMN, filled symbols indicate non-activated PMN. **(d-f) **show the corresponding curves for predicted adhesion determined by ANCOVA of logarithms (continuous line: non-activated PMN, discontinuous line: activated PMN). Under all conditions of activation decreasing shear stress increased adhesion (*P *< 0.01; ANCOVA). On maximal activated endothelium activation of PMN decreased adhesion in comparison to non-activated PMN ((b and e), *P *< 0.01, ANCOVA). On sub-maximal activated endothelium (c and f), activation of PMN also decreased adhesion in comparison to non-activated controls, especially under conditions of low shear stress (*P *< 0.01, ANCOVA).

Normal shear stress of 2 to 3 dyne/cm^2 ^prevented relevant adhesion in non-activated HUVEC-/PMN-. As expected in the model for local inflammation, maximal LPS-activation of HUVEC largely increased adhesion of non-activated PMN at 3 dyne/cm^2 ^from 42 ± 17 (HUVEC-/PMN-) to 894 ± 93 cells/mm^2 ^in HUVEC++/PMN- (*P *< 0.01, Figure 3a, b). In contrast, co-activation of PMN, in HUVEC++/PMN++, did not increase but decreased PMN adhesion by 56% when compared to HUVEC++/PMN- at 3 dyne/cm^2 ^(*P *< 0.01, Figure 3b).

At sub-maximal LPS-activation, activation of PMN in HUVEC+/PMN+ again decreased adhesion when compared to HUVEC+/PMN- (*P *< 0.01, Figure 3c). Despite persistent upregulation of CD11b this difference was most pronounced at low shear stresses where primary integrin-dependent adhesion becomes possible independent of selectin interactions [35].

According to the effect of shear stress in general, PMN adhesion increased with decreasing shear stress in all groups (Figure 3d-f). More importantly, analysis by ANCOVA showed significant interaction between cell activation and shear stress. As soon as PMN were activated, adhesion became increasingly dependent on shear stress (*P *< 0.01, Figure 3e, f).

### Relevance of selectin interactions for PMN adhesion to intact HUVEC

Addition of selectin-blocking mAbs at 2 dyne/cm^2 ^revealed that L-selectin-shedding was largely responsible for the decreased adhesion of activated PMN under normal flow (Table 2). Blocking L-selectin decreased adhesion of non-activated PMN by 30% (*P *< 0.05) down to values obtained with activated PMN whereas no effect was observed on activated PMN. Blockade of P-selectin had no significant effect in both groups, suggesting that P-selectin played no role on intact HUVEC after four hours LPS-activation. Consequently, only E-selectin remained functional under the condition of systemic hyper-inflammation and blocking the molecule in HUVEC++/PMN++ reduced adhesion down to background values observed in HUVEC-/PMN-.

**Table 2:** Effects of PMN-activation on selectin function at 2 dyne/cm^2^

	Adhesion [PMN/mm^2^]		
	
Blocking antibody	HUVEC++/PMN-	HUVEC++/PMN++	HUVEC++/PMN++/PLT++
NONE	1042 ± 61	591 ± 43	1313 ± 25
L-	744 ± 67 *	607 ± 56 ^ns vs NONE^	Ø
P-	833 ± 59 ^ns vs NONE^	596 ± 85 ^ns vs NONE^	396 ± 35 * ^vs NONE^
E-	504 ± 55 * ^vs NONE^	267 ± 32 * ^vs NONE^	Ø
L-/P-	674 ± 48 ^ns vs L-^	Ø	Ø
E-/P-	504 ± 91	230 ± 12 ^ns vs E-^	Ø
L-/E-	405 ± 59 * ^vs L-^	Ø	Ø
L-/E-/P-	343 ± 40	Ø	Ø

Adhesion in lipopolysaccharide-activated cultures (100 ng/ml; HUVEC++, PMN++, PLT++) at 2 dyne/cm^2^. Ø (not determined); * and ns (*P *< 0.05 versus indicated group or not significant, respectively). Statistical analysis with paired t-tests and correction after Bonferroni-Holm (mean ± SEM; *n *= 4).

For comparison, background adhesion in non-activated cultures (HUVEC-/PMN-) at 2 dyne/cm^2 ^revealed 247 ± 52 PMN/mm^2^.

HUVEC, human umbilical venous endothelial cells; PMN, polymorphonuclear neutrophils; PLT, platelets; L-, leukocyte selectin; P-, platelet selectin; E-, endothelial selectin; SEM, standard error of the mean.

### Effects of cell activation, shear stress and their interplay on PMN-HUVEC-rolling interactions

**Figure 4:**
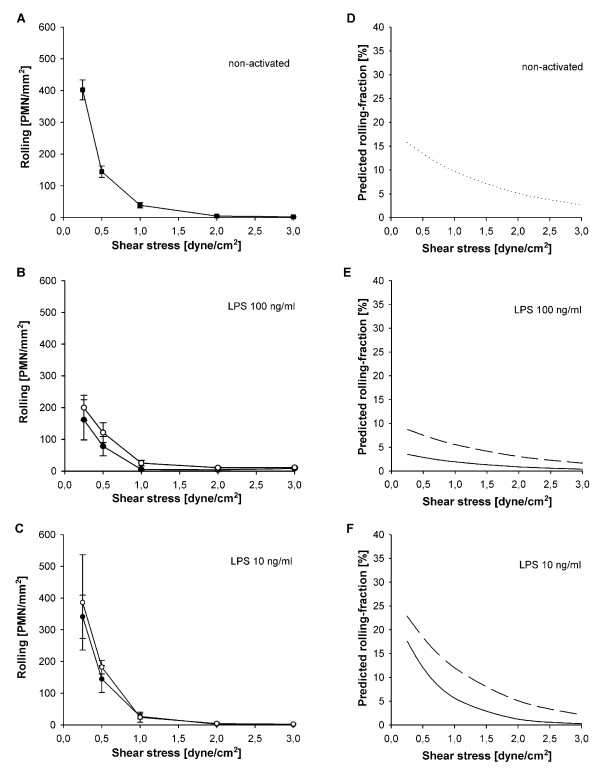
**Interdependent effects of shear stress and cell activation on PMN rolling**. Rolling of neutrophils under different activation protocols (mean ± SEM; *n *= 4), **(a) **non-activated controls, **(b) **activation with 100 ng/ml LPS and **(c) **activation with 10 ng/ml LPS. Blank symbols indicate activated PMN, filled symbols indicate non-activated PMN. **(d-f) **show the corresponding curves for predicted rolling fractions determined by ANCOVA of logarithms (continuous line: non-activated PMN, discontinuous line: activated PMN). Rolling increased with decreasing shear stress in all cultures (a-c). On non-activated (d) and sub-maximal activated HUVEC (f) decreased shear stress increased the rolling fraction (*P *< 0.05, ANCOVA) whereas it had no effect under maximal LPS-activation (e). Activation of PMN induced higher rolling fractions in comparison to non-activated PMN at sub-maximal activation ((f), *P *< 0.05, ANCOVA).

**Figure 5:**
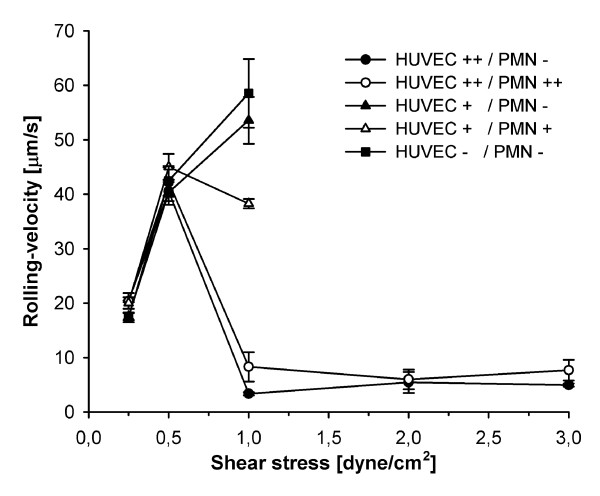
**Effects of shear stress and different conditions of activation on rolling velocities**. Plots of mean rolling velocities of >25 PMN (mean ± SEM; *n *= 4). Circles indicate maximally activated HUVEC++ (LPS 100 ng/ml) with non-activated or activated PMN (PMN- and PMN++ respectively). Triangles indicate sub-maximally activated HUVEC+ (LPS 10 ng/ml) with non-activated or activated PMN (PMN- and PMN+ respectively). Square symbols indicate non-activated controls (HUVEC-/PMN-). In non-activated and sub-maximal activated cultures without E-selectin expression, rolling PMN were too few to calculate mean velocities above 1 dyne/cm^2^. Maximal activation of HUVEC prevailed constant rolling velocities between 3 and 1 dyne/cm^2 ^characteristic for selectin-interactions. Reduction of shear stress below a critical threshold increased rolling velocities followed by a decrease with further reduction of shear stress along with the reduction in hydrodynamic flow velocity. In cultures without E-selectin markedly increased rolling velocities were observed already at 1 dyne/cm^2^.

To determine whether a dissociation of quantitative and qualitative integrin upregulation contributed to the decreased adhesion of LPS-activated PMN, rolling fractions were calculated from the number of rolling PMN in relation to total adhesion as a measure for adhesion efficiency (Figure 4). For similar reasons mean rolling velocities were calculated (Figure 5) since rolling velocity is inversely correlated with the chance of a PMN to become adherent [27].

On maximally activated HUVEC with upregulated E-selectin, PMN-activation had no influence on rolling fraction (*P *= 0.59, Figure 4e). This indicated that L-selectin shedding decreased adhesion mainly by impairing initial capture under normal shear whereas E-selectin was sufficient to translate existing rolling interactions into firm adhesion. Accordingly, E-selectin maintained slow rolling velocities above 0.5 dyne/cm^2 ^whereas markedly higher velocities were observed on HUVEC lacking E-selectin (Figure 5). Because selectin function requires the presence of shear-induced torque [36], rolling velocities increased sharply when reaching the shear-dependent threshold for E-selectin function. With further reduction in shear, rolling velocities then decreased along with the reduction in flow velocity.

On sub-maximally activated HUVEC without E-selectin, co-activated PMN showed significantly increased rolling fractions at all levels of shear stress, indicating decreased adhesion efficiency (*P *< 0.05, Figure 4f). Since HUVEC+/PMN- and HUVEC+/PMN+ differed in CD11b expression (Figure 2), the higher rolling fraction at low shear stress indicated altered qualitative integrin activation despite numerical upregulation. Accordingly, rolling velocities in HUVEC+/PMN+ equalled those that have been reported for the low-affinity configuration of β_2_-integrins [37].

### Modulation of PMN-HUVEC interactions by adherent platelets

To differentiate effects of endothelial activation from effects of endothelial injury on PMN recruitment [29-32,38] we examined the adhesion of activated PMN to platelet-covered endothelial lesions.

**Figure 6:**
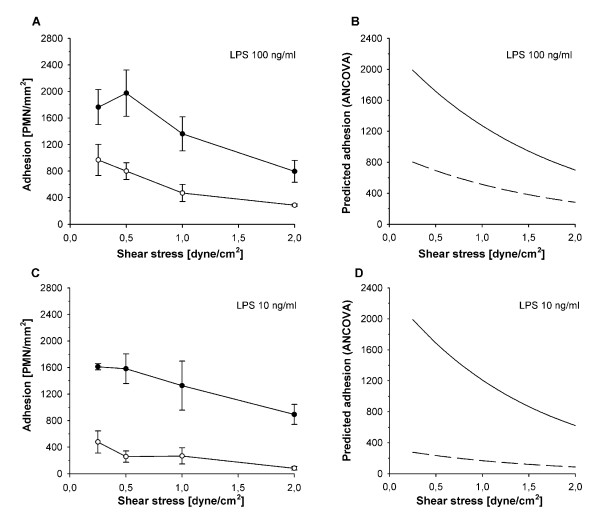
**Effects of endothelial injury, platelet interactions and shear stress on PMN adhesion**. Adhesion of activated PMN (mean ± SEM; *n *= 4) on an endothelial lesion covered with platelets (filled symbols) or intact endothelium (blank symbols) under maximal and sub-maximal activation. **(a) **activation with 100 ng/ml LPS (HUVEC++/PMN++/PLT vs. HUVEC++/PMN++), and **(c) **activation with 10 ng/ml LPS (HUVEC+/PMN+/PLT vs. HUVEC+/PMN+). **(b and d) **show the corresponding curves for predicted adhesion determined by ANCOVA of logarithms (continuous line: intact HUVEC, discontinuous line: injured HUVEC with platelets). The presence of platelets significantly increased adherence of PMN under all conditions of activation and shear, with the most pronounced effect on sub-maximally activated endothelium (*P *< 0.01; ANCOVA).

**Figure 7:**
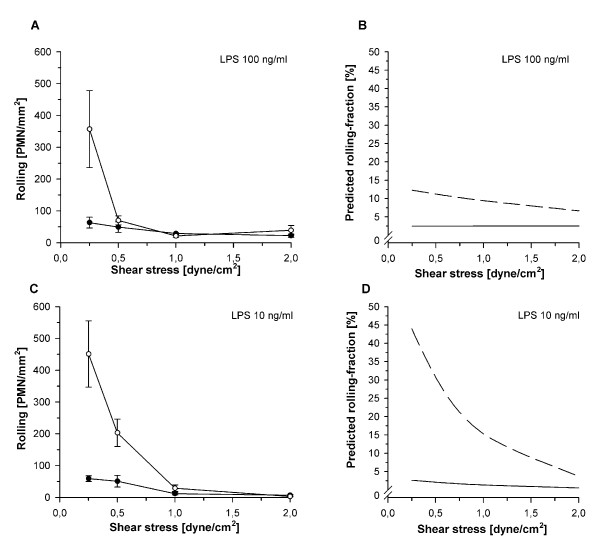
**Effects of endothelial injury, platelet interactions and shear stress on PMN rolling**. Rolling of activated PMN (mean ± SEM; *n *= 4) on an endothelial lesion covered with platelets (filled symbols) or intact endothelium (blank symbols) under maximal and sub-maximal activation. **(a) **activation with 100 ng/ml LPS (HUVEC++/PMN++/PLT vs. HUVEC++/PMN++), and **(c) **activation with 10 ng/ml LPS (HUVEC+/PMN+/PLT vs. HUVEC+/PMN+). **(b and d) **show the corresponding curves for predicted rolling-fractions determined by ANCOVA of logarithms (continuous line: intact HUVEC, discontinuous line: injured HUVEC with platelets). In contrast to intact HUVEC, rolling fractions on platelet-covered endothelial lesions remained low at both LPS concentrations and all levels of shear stress (*P *< 0.01, ANCOVA).

The presence of platelets was the strongest variable for adhesion of activated PMN. At all levels of shear stress PMN adhesion on platelet-covered, injured HUVEC increased significantly when compared to intact HUVEC (*P *< 0.01, Figure 6). At 2 dyne/cm^2 ^PMN adhesion increased 2.7-fold in maximally activated HUVEC++/PMN++/PLT (Figure 6a, b). In sub-maximally activated HUVEC+/PMN+/PLT an even larger 10-fold increase in adhesion was observed (Figure 6c, d). Additionally, platelets largely increased adhesion efficiency as documented by the consistently lower rolling fractions at both LPS concentrations and all levels of shear stress (*P *< 0.01, Figure 7). Accordingly, the rolling velocities remained low in both maximally and even sub-maximally activated co-cultures (4.5 ± 1.0 μm/s and 5.8 ± 1.5 μm/s, respectively).

Blockade of P-selectin revealed that the increased adhesion was largely due to platelet P-selectin. In contrast to its lacking effect in intact HUVEC++/PMN++, P-selectin blocking WASP12.2 decreased PMN adhesion in injured HUVEC++/PMN++/PLT by 70% (*P *< 0.01, Table 2) below the values obtained in intact HUVEC++/PMN++.

## Discussion

To provide more insight into the mechanisms that might explain the occurrence of disseminated leukocyte-related tissue damage in spite of an impaired leukocyte recruitment to local sites of inflammation during severe sepsis, we investigated the interdependent effects of cell activation, adhesion molecule expression, shear stress and a platelet-covered endothelial injury on PMN-adhesion.

In order to mimic different stages of inflammation, as they are frequently observed during the time course of severe sepsis, various constellations of PMN and endothelial activation were combined. Maximal activation of both PMN and HUVEC was considered to reflect maximal systemical inflammation in the hyper-inflammatory stage of sepsis where high concentrations of circulating mediators induce activation of leukocyte and endothelial cell adhesion molecule expression systemically throughout the circulation [2,3,10]. Submaximal activation induced upregulation of CD11b and downregulation of L-selectin on PMN to the same degree as the maximal activation did, however, without having an effect on endothelial cell adhesion molecule expression. Since this pattern of expression has been previously documented in studies on endotoxin tolerance and later hypo-inflammatory sepsis, we used the sub-maximal LPS-activation as a model for the hypo-inflammatory stage [2,3,10-17].

Apart from the different stages of inflammation, adhesion molecule expression during systemic sepsis differs from local inflammation in another important aspect. In local inflammation upregulation of leukocyte integrins and shedding of L-selectin does not occur before entering the inflamed tissue [1]. To account for this difference, activated HUVEC were used in combination with non-activated PMN to mimic local inflammation whereas PMN were treated with the same LPS concentrations as HUVEC to model sepsis-associated systemic inflammation.

The results demonstrate that impaired recruitment of systemically activated PMN to local sites of inflammation during severe sepsis [2,3,8-10] can be explained by two mechanisms. At normal shear stress, shedding of L-selectin reduced adhesion in our experiments by impairing initial capture. With reduction in shear stress this mechanism became less important and adhesion increased. However, adhesion of activated PMN still appeared reduced in comparison to non-activated PMN. This reduction was most obvious in the sub-maximally activated group at shear stresses where primary integrin-dependent adhesion occurs independently of selectin interactions [35,36]. Since CD11b remained upregulated on sub-maximally activated PMN, this finding indicates a dissociated quantitative and qualitative integrin-activation as the second mechanism for altered adhesion of activated PMN. Integrin-dependent adhesion involves a cooperative and sequential process of LFA-1-dependent initiation and Mac-1-dependent stabilization [39]. The increased integrin-affinity, necessary to form bonds with their endothelial ligands, is transient within minutes after activation [40]. Accordingly, we observed decreased integrin-dependent adhesion efficiency after PMN-activation and the rolling velocities equalled those that have been reported for the low affinity configuration of LFA-1 [37].

Reflecting the well-known inverse correlation of shear stress and adhesion in general [19-22] PMN-adhesion was largely influenced by shear stress in all cultures. More importantly, the net effect of shear stress depended on the inflammatory state of the interacting cell populations. Firm adhesion of non-activated PMN to maximally activated HUVEC showed the smallest susceptibility to shear stress, which seems reasonable for targeting leukocytes to a local site of inflammation independent of variations in postcapillary blood flow. As soon as the PMN were activated, loss of L-selectin rendered cell interactions increasingly susceptible to shear stress. In sub-maximally activated cultures, shear stress became the prevailing determinant of PMN adhesion. Regarding the heavily decreased flow velocities that may arise in small vessels of the septic microcirculation even when macrohemodynamics have been restored [23-25], this finding suggests that variations in shear stress largely influence leukocyte accumulation once systemic inflammation has evolved. Additionally, their influence seems to increase as soon as hyper-inflammation has turned into hypo-inflammation as might occur early, especially in those patients with poor prognosis [12,13].

Far exceeding the effects of shear stress is the platelet-covered endothelial lesion, which proved to be the strongest determinant of PMN-adhesion at all levels of shear stress. In maximally activated cultures, PLT-PMN interactions increased PMN adhesion by two-fold. At the sub-maximal LPS dose, an even more dramatic 10-fold increase was observed. Both findings indicate that endothelial cell damage gains a leading role for the spatial distribution of leukocyte accumulation through PLT-PMN interactions under conditions of systemic leukocyte activation and becomes exceedingly pronounced when true endothelial cell activation is outweighed by endothelial cell damage, as might occur in the hypo-inflammatory stage of severe sepsis [11-17,30,32]. At sites of endothelial cell injury, platelet activation occurs through contact to the subendothelial matrix and does not become altered when endothelial cell activation is impaired [34,38]. Platelet adhesion to the intact endothelium, in contrast, requires the presence of endothelium-derived P-selectin [34]. Although the latter mechanism contributes to leukocyte accumulation in rodents, humans and primates are not able to sustain endothelial P-selectin expression beyond the very first minutes of inflammation because of a lack in transcriptional regulation [34,41]. Accordingly, blocking P-selectin had no effect on PMN-adhesion to intact HUVEC after four hours LPS-activation in our human adhesion experiments.

Independent from endothelial cell activation platelet-covered lesions provide a rich source of platelet-derived P-selectin [33,34]. In our experiments the high density of platelet- but not endothelium-derived P-selectin largely increased adhesion and adhesion efficiency as reflected by the different effect of P-selectin blockade on intact and injured HUVEC. Even in rodents, who are able to sustain endothelial P-selectin expression for a longer time than humans [34,41], platelet but not endothelial P-selectin contributes to leukocyte-related organ dysfunction during severe inflammation [42-44]. In contrast to a previous study that interpreted adhesion of leukocytes from septic individuals to a platelet surface as a general sign for increased leukocyte adhesiveness during sepsis [45], we, therefore, considered PMN adhesion to the platelet-covered subendothelial matrix as a model for leukocyte accumulation in the injured, rather than the activated, but intact microvasculature in a source of infection.

Since the effects of shear stress, tissue hypoxia, cell activation and cell injury are hardly distinguishable from each other during sepsis *in vivo *and, in part, are species-related, we decided to use a flow chamber to examine their interplay in a human setting. Clearly, this simplified *in vitro *model has other inherent limitations since it neither includes true infection nor simulates all aspects of sepsis in an intact organism. For instance, we had to abstain from inducing true endotoxin tolerance since this would have required prolonged cell culture with inevitable confounding effects on adhesion molecule expression in an otherwise comparative experimental setting. Additionally, the use of cell suspensions instead of whole blood influences rheological properties and the fixed diameter of the flow channel precludes effects of luminal narrowing that may arise in small vessels during leukocyte adhesion. Apart from directly favouring further adhesion, these effects may also influence cell interactions *in vivo *by decreasing blood flow and oxygen transport.

As a necessary simplification instead, we used different LPS-concentrations and standardized reproducible hydrodynamic conditions in an otherwise unchanged comparative model to investigate the mechanisms of leukocyte accumulation during different stages of systemic inflammation. Although this model is artificial in many aspects, flow chamber experiments have proven valid for studying cell interactions in a number of studies including direct comparison with leukocyte adhesion in animals [26,46]. Additionally, the experimental model resulted in adhesion molecule patterns as they have been observed under different stages of sepsis-associated systemic inflammation *in vivo *[2,3,10,12-17].

## Conclusions

In summary, our findings indicate a maldistribution of systemically activated leukocytes away from sites of local inflammation with intact endothelium and normal blood flow towards sites with compromised perfusion or endothelial cell injury. Because of L-selectin shedding and altered integrin function, this maldistribution might occur even during the early hyper-inflammatory stage. It seems to become exceedingly pronounced, however, when endothelial LPS sensitivity is decreased, as might occur in patients with hypo-inflammatory cytokine profiles [12-16]. From a clinical perspective, this suggests that hemodynamic resuscitation should not only be targeted to increase oxygen delivery during the first hours of sepsis but to normalize microvascular blood flow velocity as an option to prevent disseminated leukocyte accumulation throughout the course of the syndrome. Regarding the role of platelets, our observations add a further piece to the puzzle of platelet-neutrophil interactions during severe inflammation. In addition to those studies that have documented a contributory role for platelets in leukocyte-related tissue damage [42-44], our results suggest that they might gain a leading role as soon as endothelial damage outweighs endothelial activation. Tailoring the various forms of anti-platelet therapies to sepsis stage and immune balance may therefore represent a promising approach to increase their effectiveness in the future.

## Key messages

• Activation of leukocytes renders adhesion increasingly susceptible to shear stress.

• Presence of a platelet-covered endothelial injury overcomes this effect and seems to become the prevailing factor for leukocyte accumulation under the condition of systemic inflammation.

• Together these mechanisms favor the maldistribution of leukocytes away from local sources of inflammation and towards areas with compromised flow and/or endothelial damage.

## Abbreviations

ANOVA: analysis of variance; ANCOVA: analysis of covariance; E-selectin: endothelial selectin; HUVEC: human umbilical venous endothelial cells; ICAM-1: intercellular adhesion molecule-1; IL-10: interleukin-10; LFA-1: lymphocyte function antigen-1; LPS: lipopolysaccharide; L-selectin: leukocyte selectin; mAb: monoclonal antibody; MAC-1: macrophage antigen-1; MFI: median of fluorescence intensity; PLT: platelets; PMN: polymorhponuclear neutrophils; P-selectin: platelet selectin; SEM: standard error of the mean; TNF-α: tumor necrosis factor-α.

## Competing interests

The authors declare that they have no competing interests.

## Authors' contributions

AP and BN conceived of the study, participated in its design and coordination, and drafted the manuscript. VS and AH carried out the adhesion assays. JR and HAH participated in the design of the study and helped to draft the manuscript.
